# Benign prostatic enlargement can be influenced by metabolic profile: results of a multicenter prospective study

**DOI:** 10.1186/s12894-017-0211-9

**Published:** 2017-04-04

**Authors:** Mauro Gacci, Arcangelo Sebastianelli, Matteo Salvi, Cosimo De Nunzio, Linda Vignozzi, Giovanni Corona, Tommaso Jaeger, Tommaso Chini, Giorgio Ivan Russo, Mario Maggi, Giuseppe Morgia, Andrea Tubaro, Marco Carini, Sergio Serni

**Affiliations:** 1 Department of Urology, University of Florence, Careggi Hospital, Florence, Italy; 2 Department of Urology, Sant’Andrea Hospital, University “La Sapienza”, Rome, Italy; 3 Department of Clinical Physiopathology, University of Florence, Florence, Italy; 4 Endocrinology Unit, Maggiore-Bellaria Hospital, Bologna, Italy; 5 Department of Urology, Policlinico Hospital, University of Catania, Catania, Italy

**Keywords:** Benign prostatic enlargement, Benign prostatic hyperplasia, Lower urinary tract symptoms, Metabolic syndrome, Dyslipidemia

## Abstract

**Background:**

In last years Metabolic Syndrome (MetS) has been closely associated to Benign Prostatic Enlargement (BPE) Aim of our study is to evaluate the effect of MetS and each single MetS parameter on prostate growth in men surgically treated for BPE.

**Methods:**

Overall, 379 men were prospectively enrolled in two tertiary referral centers. *Calculated prostate volume* (PV) was measured with transrectal US defining the antero-posterior (AP), the cranio-caudal (CC) and the latero-lateral (LL) diameters through the ellipsoid formula, while *raw PV* was calculated by suprapubic US. MetS was defined according to the NCEP-ATPIII criteria.

**Results:**

One-hundred and forty men (36.9%) were affected by MetS. The number of MetS parameters (0 to 5) and the presence of MetS were correlated with the *calculated PV*. The number of MetS parameters were also directly related to increasing prostate diameters. At the binary logistic regression, MetS resulted associated to high (>60 cc) raw and calculated PV. Moreover, multivariate analysis suggested that AP diameter was mainly correlated with HDL cholesterol (r:-0.3103, *p* = 0.002) CC diameter with triglycerides (r:-0.191, *p* = 0.050) and LL diameter with systolic blood pressure (r:0.154, *p* = 0.044). However, at the binary logistic regression, only low HDL Cholesterol was the main determinant for the enlargement of all diameters and consequently of the whole PV.

**Conclusions:**

Metabolic factors, specially dyslipidemia, could play a central role in the pathogenesis and progression of BPE/LUTS. Interventional studies are needed to evaluate the impact of early treatment of dyslipidemia on progression of LUTS/BPH.

## Background

Benign prostatic hyperplasia (BPH) is one of the most common conditions among middle and advance-aged men [1]. Autopsy studies revealed presence of BPH in 42% of men aged 51–60 year and 85% among men older than 80 year; BPH is characterized by stromal and cell hyperplasia which can lead to the development of prostatic bladder outlet obstruction (BOO) and Lower Urinary Tract Symptoms (LUTS); severe BPH leads to deterioration of QoL and has relevant socio-economic costs [2]. Historically BPH pathogenesis is linked to age and androgens effect but more recently other factors including family history, ethnicity, lifestyle behaviours (reduced physical activity, cigarette smoking and high fat diet) as well as metabolic diseases have been suggested to play an important role [3, 4].

Metabolic syndrome(MetS) is a worldwide complex disorder with high socioeconomic impact. MetS describes the combination of several metabolic abnormalities, including central obesity, hypertension, dyslipidemia, insulin resistance with compensatory hyperinsulinemia, and glucose intolerance [5].

In the last 15 years several MetS components have been closely associated with BPH, suggesting that MetS has very heterogeneous clinical ramifications [6–8].

Although the relationship between BPH/LUTS and MetS is still poorly understood, some findings suggest that men with metabolic alterations faster develop [6] BPH or are more likely to undergo BPH surgery, [7] supporting the hypothesis that pathological alterations typical of MetS also predispose to the development and progression of BPH/LUTS. Indeed, in a recent meta-analysis, we demonstrated that subjects with MetS have significantly higher total and transitional zone prostate volume [9].

Aim of the present study is to evaluate the correlations between the presence of MetS and each single MetS parameter on prostate’s anthropometric measures in men surgically treated for BPE.

## Methods

### Study population and design

Between January 2012 and September 2013, 379 consecutive patients undergone prostatectomy for LUTS due to large BPE, were prospectively enrolled in two tertiary referral centers. In both high volume referral centers, all patients included in this trial were managed by surgeons skilled in diagnosis and treatment of LUTS/BPE. Informed consent for the study was obtained from participants. The study did not require any deviation of the Good Clinical Practice so was conducted in accordance with the principles expressed in the Declaration of Helsinki.

In the study were included patients undergone simple open prostatectomy (OP) or transurethral resection of the prostate (TURP) for moderate to severe LUTS due to BPE refractory to medical treatment. Patients with previous history of prostate surgery, chronic medication for prostatitis and/or urinary infection or bladder stone or known malignant disease including prostate cancer were excluded.

PSA values and prostate volume were evaluated during the pre-hospitalization visits. *Raw prostate volume* was calculated by suprapubic US (by using the “estimated ellipsoid volume” based on prostatic circumference), while *calculated prostate volume* was measured by transrectal US defining the antero-posterior (AP), the cranio-caudal (CC) and the latero-lateral (LL) diameters through the ellipsoid formula (D1xD2xD3xπ/6). OP and TURP were performed as previously reported [10, 11]. LUTS were measured by the International Prostate Symptom Score (IPSS) and categorized as storage and voiding symptoms, immediately before surgery and 6 to 12 months postoperatively.

### Definition of MetS

MetS was defined according to criteria defined by the National Cholesterol Education Program-Third Adult Treatment Panel (NCEP-ATPIII) [5, 12]. According these criteria MetS is defined by the presence of at least 3 of the following parameters: (1) waist circumference >102 cm; (2) triglycerides ≥150 mg/dl or treatment for hypetriglyceridemia, (3) HDL-Co < 40 mg/dl or treatment for reduced HDL-C, (4) blood pressure ≥ 130/85 mmHg or current use of antihypertensive medications, and (5) fasting blood glucose >110 mg/dl or previous diagnosis of type 2 diabetes mellitus. All these items of MetS were considered individually (single parameters above vs below cut-off points), as sum of continuous variables (one if the single parameter is positive for MetS, zero if the single parameter is negative), and combined according to MetS (present or absent).

### Statistical analyses

Unpaired two-sided Student’s t tests has been used for comparisons between men with or without MetS, to compare normally distributed parameters; in all other cases, Mann-Whitney *U* test has been used. Correlations have been assessed using Pearson’s or Spearman’s method for normally or non-normally distributed data.

Moreover, we included significant data in a binary logistic model regression to calculate the main determinant of both *raw* and *calculated* prostate volume.

All the analyses were obtained with SPSS statistics 20.0 version for windows XP and a p <0.05 was considered statistically significant.

## Results

Three-hundred seventy-nine non selected consecutive men undergone surgical treatment of BPH were recruited in two tertiary referral centers. One-hundred and forty men (36.9%) were affected by MetS: preoperative patient’s characteristics, stratified according to MetS diagnosis, are reported in Table 1.

**Table 1:** Descriptive statistics of population of men included in the study, stratified according to their MetS profile

Patients (*n* = 379)		With MetS (*n* = 140)	Without MetS (*n* = 239)	
		Mean ± SD	Mean ± SD	*p* value
Demographic	Age (years)	70.0 ± 7.4	68.5 ± 8.8	0.059
	BMI (Kg/m^2^)	27.5 ± 3.5	25.8 ± 2.4	0.000
	Smokers, Number, (%)	108 (77.1%)	171 (71.5%)	0.417
Prostate Features	Prostate Volume (cc)	88.9 ± 59.1	77.8 ± 41.2	0.053
	PSA (ng/mL)	3.9 ± 3.7	3.0 ± 3.2	0.062
Prostate treatment	α-blockers, Number, (%)	103 (73.5%)	164 (68.6%)	0.200
	5-ARI, Number, (%)	23 (16.4%)	33 (13.8%)	0.467
MetS parameters	WC	104.6 ± 12.9	97.2 ± 7.3	0.000
	Systolic BP	134.9 ± 14.7	131.3 ± 14.6	0.016
	Diastolic BP	78.7 ± 8.5	76.7 ± 8.0	0.020
	Glycemia	108.8 ± 37.1	94.1 ± 16.3	0.000
	Triglyceride	149.8 ± 54.3	111.2 ± 42.7	0.000
	HDL Cholesterol	41.5 ± 11.0	49.1 ± 7.4	0.000

At univariate analysis *raw prostate volume* resulted statistically related with systolic blood pressure and serum trygliceride levels (r = 0.114, *p* = 0.035 and r = 0.126, *p* = 0.013 respectively), while *calculated prostate volume* resulted related with systolic blood pressure, serum trygliceride levels and serum HDL levels (r = 0.179, *p* = 0.015 and r = 0.279, *p* < 0.001 and r = -0.303, *p* = *p* < 0.001 respectively). The number of metabolic syndrome parameters (0 to 5) and the presence of MetS (≥3/5 parameters) were significantly correlated with the *calculated prostate volume* (r = 0.244, *p* = 0.001 and r = 0.284, *p* < 0.001, respectively). At age-adjusted multivariate analyses, systolic blood pressure, serum HDL levels and the number of MetS parameters were still statistically significantly correlated to *calculated prostate volume* (r = 0.175, *p* = 0.014, r = -0.256, *p* = 0.004 and r = 0.202, *p* = 0.007 respectively).

At the binary logistic regression (Table 2) considering all the main determinants of prostate volume, including age, BMI and use of 5-alpha-reductase inhibitors, MetS resulted a statistically significant risk factor for large (>60 cc) raw and calculated prostate volume (OR: 2.43 [95% CI: 1.444.09), *p* = 0.001 and OR: 4.28 [95% CI: 2.15–8.52), p < 0.001, respectively). A similar data was obtained by using the median (>70 cc) raw volume (OR: 1.82 [95% CI: 1.08–3.09), *p* = 0.026).

**Table 2:** Binary logistic regression based on prostate volume ≥ 60 cc vs. prostate volume < 60 cc. *Age (< 65* vs. *≥ 65), BMI (< 25 kg/m* ^*2*^ vs. *≥ 25 kg/m* ^*2*^ *), Use of 5 ARI (no* vs. *yes), Presence of MetS (no* vs. *yes). OR* Odds ratio. *LL* Lower Limit. *UL* Upper Limit

	OR	LL 95% CI for OR	UL 95% CI for OR	*P* value
RAW Prostate volume (*N* = 379)				
Age	0.995	0.962	1.029	0.769
BMI	0.936	0.859	1.021	0.136
Use of 5ARI	1.054	0.541	2.056	0.877
Presence of MetS	2.430	1.441	4.095	*0.001*
CALCULATED Prostate volume (*N* = 187)				
Age	0.972	0.930	1.015	0.200
BMI	0.854	0.760	0.959	*0.008*
Use of 5ARI	1.304	0.625	2.719	0.479
Presence of MetS	4.278	2.149	8.519	*0.035*

Italic=statistically significant

**Fig. 1:**
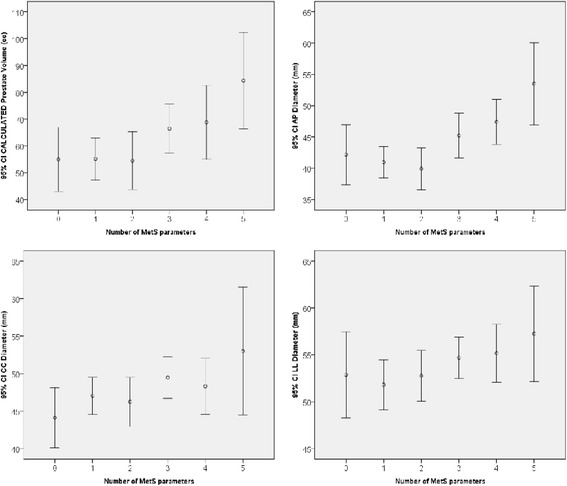
Mean and 95% confidence interval of the mean of calculated prostate volume, antero-posterior (AP), cranio-caudal (CC) and latero-lateral (LL) diameters, stratified according to the number of MetS parameters

The number of MetS parameters, resulted directly related with the calculated prostate volume (r = 0.244, *p* = 0.001), with the antero-posterior (r = 0.231, *p* = 0.002), the cranio-caudal (r = 0.192, *p* = 0.009) and the latero-lateral diameter (r = 0.171, *p* = 0.020, see Fig. 1). At the age-adjusted multivariate analysis, including all the diameters, only the AP diameter was significantly related with the number of MetS parameters (r = 2.266, *p* = 0.025).

**Fig. 2:**
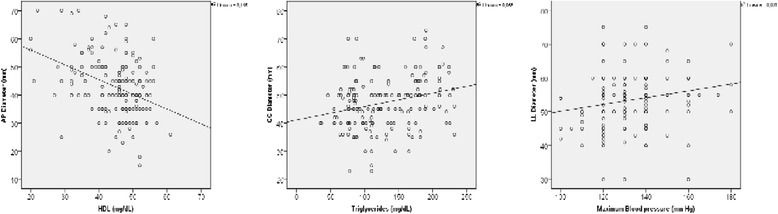
Scatterplot diagram of correlation between AP diameter and HDL Cholesterol, CC diameter and triglyceride, LL diameter and Systolic blood pressure

**Fig. 3:**
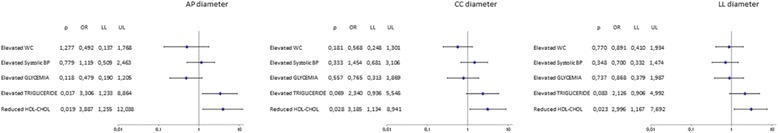
Odds Ratio (OR) based on based on the median prostate diameters (AP = 40 mm, CC = 45 mm, LL = 55 mm) as derived from a logistic regression model adjusted for: Age, PSA, smoking, consumption of finasteride, presence of MetS. *p* = Pvalue. OR = Odds ratio. LL = Lower Limit. UL = Upper Limit

Furthermore, at the multivariate analysis based on significant parameters that can influence prostatic growth, the AP diameter was mainly correlated with HDL cholesterol (adjusted r for age, BMI and 5-ARIs: -0.3103, p = 0.002, see Fig. 2a), the CC diameter with triglycerides (adjusted r for age, BMI and 5-ARIs: -0.191, *p* = 0.050, see Fig. 2b) and the LL diameter with systolic blood pressure (adjusted r for age, BMI and 5-ARIs: 0.154, *p* = 0.044, see Fig. 2c). However, the binary logistic regression based on a median prostate diameters (AP = 40 mm, CC = 45 mm, LL = 55 mm) adjusted for age, presence of MetS, cigarette smoking and assumption of 5ARI, demonstrated that low HDL Cholesterol was the main determinant for the enlargements of all diameters and consequently of the whole prostate volume (see Fig. 3).

## Discussion

Metabolic syndrome (MetS) is a cluster of cardiovascular and metabolic risk factors, associated with insulin resistance [5]. For first in 1998 Hammarsten et al. [13] described the possible relationship between some components of MetS and BPH. In their study, annual transitional prostate volume (TPV) growth rate was significantly higher in BPH patients with MetS as compared with those without MetS (1.019 ml/yr vs 0.699 ml/yr, respectively). After this preliminary work, several authors have documented a possible association between MetS and BPH [14–16] but other authors didn’t confirm this association [17]. Interestingly in a meta-analysis of the available evidence we found that subjects with MetS had significantly higher total prostate volume when compared to those without MetS (+1.8 [95% CI: 0.74;2.87] ml; *p* < 0.001) and these datas are in agreement to the present one. The number of metabolic syndrome parameters (1 to 5) and the presence of MetS itself were related with the prostate diameters as well as *calculated prostate volume*, supporting a positive role for metabolic derangements in the progression of BPE.

The pathogenetic mechanisms underlying the association between MetS and BPH/LUTS are not completely understood. Either clinical or experimental evidence supports the role of chronic inflammation as possible link [18]. Although it has been known for at least 30 years that inflammation directly or indirectly contributes to prostate overgrowth, the role of impaired immunoresponse in BPH pathogenesis has been recently accepted [18].

The effect of MetS to BPH pathogenesis probably starts in early adulthood. Indeed, in a previous study on a population of 222 relatively young men seeking medical care for couple infertility, we found a significant association among increasing BMI, higher prostate volume and several sonographic features of prostate inflammation [19]. In addition, higher BMI was significantly related to higher value of IL-8 in seminal vesicle tissues, a reliable surrogate marker of prostate inflammatory diseases [20]. In the same population we also found that MetS severity was associated with increased prostate volume [21]. This association indicates that the effect of MetS on prostate growth begins very early and is detectable even in young adulthood.

We recently developed a non-genomic animal model of MetS, by exposing rabbits to a high-fat diet (HFD) for twelve weeks [22]. Accordingly to the aforementioned epidemiological clinical datas, severe prostatitis-like syndrome, tissue remodeling [22, 23] and bladder dysfunction [22] were demonstrated in animal models of MetS rabbits. Infiltration of inflammatory cells and fibrosis were observed in prostate of MetS rabbits [24]. In addition, we recently demonstrated the capacity of human myofibroblast prostatic cells to secrete several inflammatory cytokines and chemokines, including IL-8, in response to oxidized LDL (oxLDL) and insulin [25, 26]. These datas indicate that different MetS features, mainly dyslipidemia (oxLDL) and insulin resistance, could boost inflammation and tissue-remodelling in BPH. Indeed in a multicentre study on 271 consecutive men treated with simple prostatectomy, the presence of MetS (in particular MetS-associated dyslipidaemia) was associated with more severe intraprostatic inflammation [27, 28]. Among MetS components, reduced HDL cholesterol and elevated triglycerides were significantly associated with elevated prostate inflammatory score (IS) and CD45 positivity. According to these datas, the present article shows that reduced HDL cholesterol levels were inversely related to all prostatic diameters. Dyslipidemia could have a detrimental effect on prostate cells, boosting prostate inflammation, a key factor in the development and progression of BPH/LUTS. Interestingly, a retrospective population-based cohort study on 2447 men aged 40–79 years, showed that statins assumption was associated with a 6.5 to 7-years delay in the new onset of moderate/severe LUTS/BPE [29]. Similarly, longitudinal datas from Health Professionals Follow up Study (HPFS), a prospective database on more than 18,000 US men, demostrated that men with higher total and abdominal adiposity or who gained weight were more likely to develop LUTS or experience progressive LUTS [30].

Our prospective study has several limitations. Firstly, we included men treated exclusively in two tertiary referral centers for BPH surgery: this population of men with large prostate (≥80 cc), and marked reduction of urinary flow parameters (Qmax < 9 mL/sec) may be very different to that of the general community. Then, we didn’t adjust our datas for additional parameters such as physical activity. Finally, we had data on calculated prostate volume only for 187 patients.

## Conclusions

In conclusion, present data along with recent evidences, suggest that metabolic factors could play a crucial role in the pathogenesis of LUTS/BPH. Further interventional studies are needed to prove the potential effect of dyslipidemia treatment on LUTS/BPH, and in particular on prostate enlargement.

## Abbreviations

BPH: Benign prostatic hyperplasia
BOO: Bladder outlet obstruction
LUTS: Lower urinary tract symptoms
MetS: Metabolic syndrome
OP: Open prostatectomy
TURP: Transurethral resection of the prostate
